# Surgical Management of a Painful Temporal Bone En Plaque Meningioma: A Case Report and Review of the Literature

**DOI:** 10.1055/a-2625-9498

**Published:** 2025-06-16

**Authors:** Prishae Wilson, Alok A. Bhatt, Mark A. Edgar, Alfredo Quiñones-Hinojosa, João Paulo Almeida, Mallory Raymond

**Affiliations:** 1 Department of Otolaryngology—Head and Neck Surgery, Mayo Clinic in Florida, Jacksonville, Florida, United States; 2 Department of Radiology, Mayo Clinic in Florida, Jacksonville, Florida, United States; 3 Department of Laboratory Medicine and Pathology, Mayo Clinic in Florida, Jacksonville, Florida, United States; 4 Department of Neurosurgery, Mayo Clinic in Florida, Jacksonville, Florida, United States; 5 Department of Neurosurgery, Indiana University, Indianapolis, Indiana, United States

**Keywords:** case report, temporal bone, en plaque meningioma, pain, management

## Abstract

Temporal bone en plaque meningiomas can present management challenges, particularly when accompanied by severe pain. We report the case of a 42-year-old woman who was initially diagnosed with chronic otitis media but was later found to have a painful left temporal bone en plaque meningioma. Despite conservative therapy, her pain progressed, prompting the decision to undergo surgical resection, which resulted in considerable pain relief. This report illustrates the influence of pain on surgical decision-making for temporal bone en plaque meningiomas and reviews the literature on their variable presentations and management strategies.

## Introduction

Meningiomas are the most prevalent benign intracranial neoplasms, classified as either en masse or en plaque. 1 In contrast to the more prevalent en masse subtype, the en plaque variant, which makes up 2 to 9% of all meningiomas, is characterized by dural invasion, thickening, and infiltration of surrounding bone. En plaque meningiomas most commonly occur in the spheno-orbital region but may also occur along the cerebral convexity, temporal bone, and foramen magnum. 2

While challenges in diagnosing and managing en plaque meningiomas are well documented, the role of pain in guiding surgical decisions remains underexplored despite its potential to significantly impact quality of life. Although pain is not commonly reported across all en plaque meningiomas, it may occur more frequently in lesions involving the temporal bone. Specific incidence data on pain are limited, but in a series of 36 patients with primary ear and temporal bone meningiomas of various subtypes, approximately 14% reported pain. 3 4 5 Involvement of otologic and adjacent neurovascular structures may contribute to symptoms such as otalgia, aural fullness, and headaches, which are often difficult to manage and show variable response to surgical intervention.

This report highlights a patient with refractory chronic otitis media (COM) and worsening left-sided headaches who was ultimately diagnosed with a left temporal bone en plaque meningioma and experienced significant pain improvement following surgical resection. Additionally, we conducted a comprehensive review of the literature on documented cases of temporal bone en plaque meningiomas, including their clinical manifestations and treatment strategies.

## Case Report

**Fig. 1:**
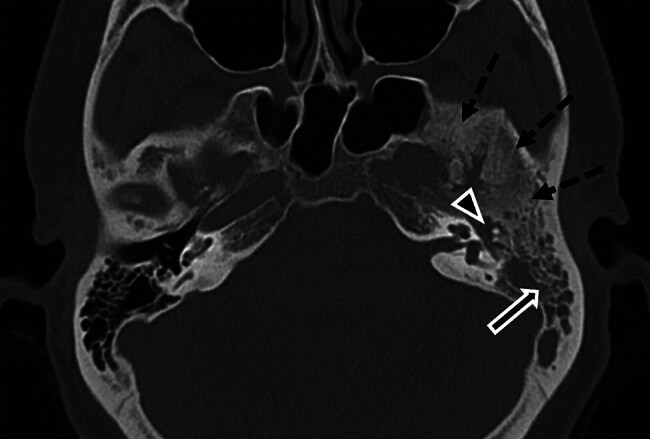
Axial computed tomography (CT) image through the bilateral temporal bones demonstrates trapped fluid within the left mastoid air cells (solid arrow), soft tissue attenuation within the middle ear cavity (arrowhead), and diffuse sclerosis and hyperostosis of the left lateral and middle skull base (dashed arrows). Note the normal right side.

**Fig. 2:**
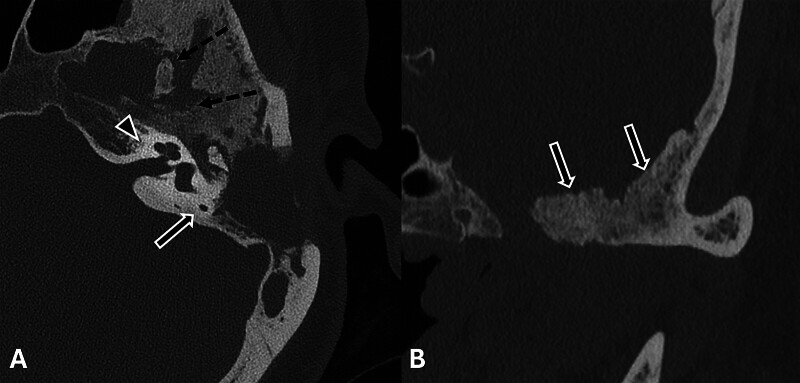
( **A** ) Axial computed tomography (CT) of the left temporal bone demonstrates mastoidectomy (solid arrow), persistent soft tissue attenuation within the middle ear cavity (arrowhead), and unchanged sclerosis and hyperostosis of the left middle and lateral skull base (dashed arrows). ( **B** ) Coronal CT demonstrates hyperostosis of the left middle skull base (arrows).

**Fig. 3:**
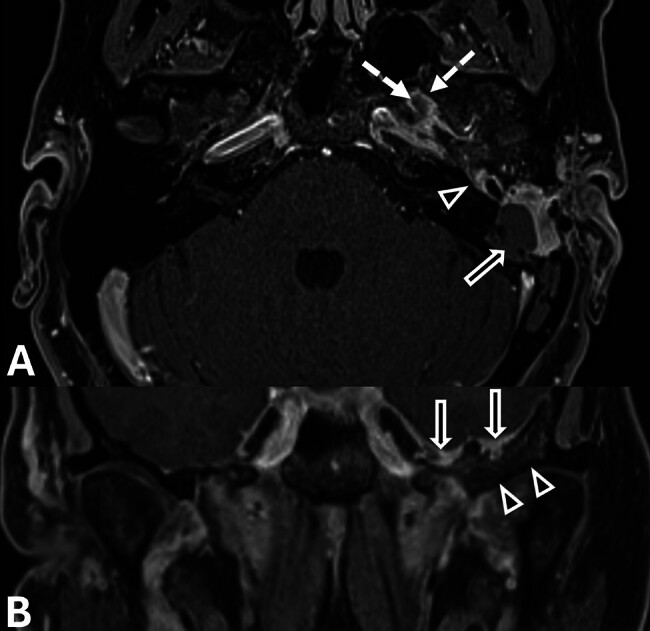
( **A** ) Axial T1-weighted postcontrast fat-saturated image demonstrates mastoidectomy bowl (solid arrow), abnormal enhancement in the middle ear cavity (arrowhead), and along the floor of the left middle cranial fossa (dashed arrow) adjacent to the hyperostosis. ( **B** ) Coronal T1-weighted postcontrast fat-saturated image demonstrates abnormal soft tissue enhancement along the floor of the left middle cranial fossa (arrows) and hyperostosis of the left middle skull base (arrowheads). Note the normal magnetic resonance imaging appearance of the right side.

A 42-year-old woman with a history of COM presented for evaluation of worsening left-sided hearing loss, otalgia, pulsatile tinnitus, and facial numbness. She described a 6-year history of COM, initially treated with seven tympanostomy tubes, topical and oral antibiotics, and an eventual tympanomastoidectomy that resolved the otorrhea but did not improve her hearing or aural fullness. Otoscopic examination showed a narrowed but patent external auditory canal and a severely thickened, hypervascular tympanic membrane. Audiometric testing revealed left-sided mixed hearing loss with a 35-decibel (dB) air–bone gap (ABG) and a word recognition score (WRS) of 72%. A preoperative computed tomography (CT) scan from an outside facility ( Fig. 1 ) was compared with an updated postoperative CT ( Fig. 2 ), which demonstrated progressive hyperostosis of the external auditory canal, septa within the mastoid air cells, and the central skull base. Magnetic resonance imaging (MRI) showed adjacent thickened dura, suggesting reactive changes related to an underlying cholesteatoma, fibrous dysplasia, or an intraosseous meningioma ( Fig. 3 ).

**Fig. 4:**
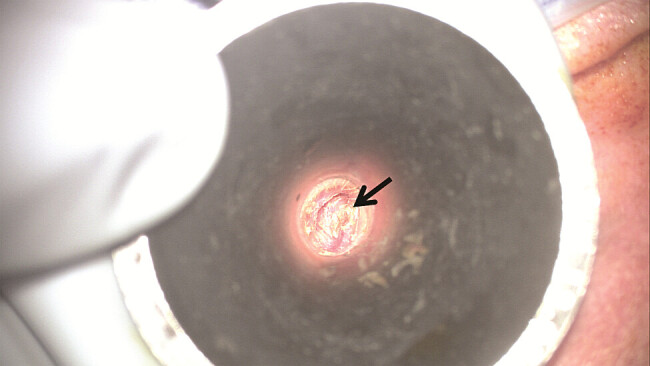
Preoperative photograph of the posterior aspect of the left external auditory canal taken through a speculum, demonstrating significant narrowing, inflammation, and evidence of neo-osteogenesis (arrow).

**Fig. 5:**
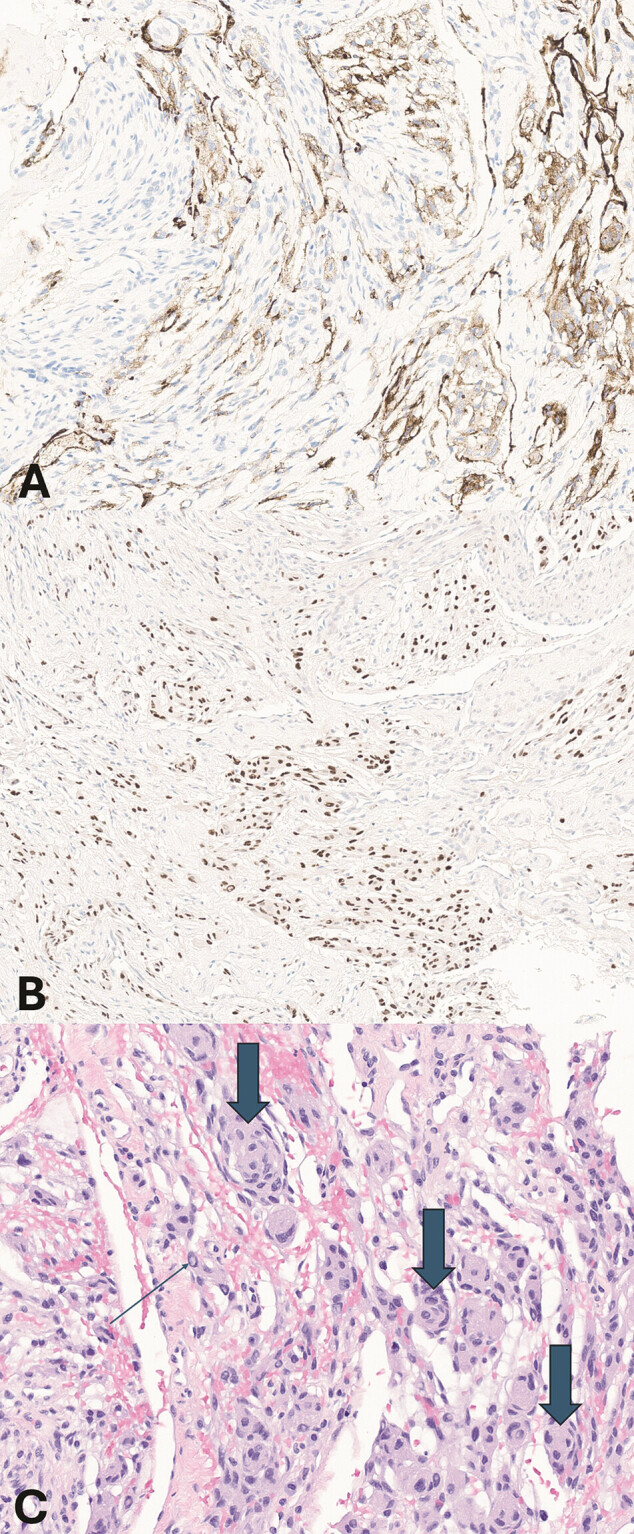
( **A** ) Epithelial membrane antigen (EMA) immunostaining reveals predominantly membranous staining characteristic of meningothelial cells. ( **B** ) Progesterone receptor (PR) immunostaining demonstrates nuclear expression. ( **C** ) Hematoxylin and eosin (H&E) staining shows cellular whorls (large arrows) and cells with intranuclear cytoplasmic pseudoinclusions (small arrow).

With symptoms worsening and new imaging suggesting an intraosseous meningioma, she underwent revision left tympanoplasty, mastoidectomy, and ossicular chain reconstruction to obtain a diagnosis and attempt to reestablish middle ear function. Intraoperative findings included extensive inflammation in the middle ear and mastoid, significant neo-osteogenesis and osteitis affecting the ear canal ( Fig. 4 ), mastoid cavity rim, tegmen mastoideum, and tegmen tympani, as well as fixation of the malleus and incus. Pathologic evaluation of soft tissue biopsies centered over the epitympanum and bone just inferior to the tegmen tympani revealed nonspecific inflammation. Immunohistochemical staining demonstrated a population of epithelial membrane antigen-positive cells ( Fig. 5A ) and S100- and SOX10-negative cells. Progesterone receptor (PR) immunostaining showed nuclear expression ( Fig. 5B ), and hematoxylin and eosin staining revealed cellular whorls and intranuclear cytoplasmic pseudoinclusions ( Fig. 5C ), findings consistent with intraosseous meningioma. Postoperative audiometric testing at that time showed an air pure-tone average (PTA) of 51 dB, an ABG of 33 dB, and a WRS of 100%.

**Fig. 6:**
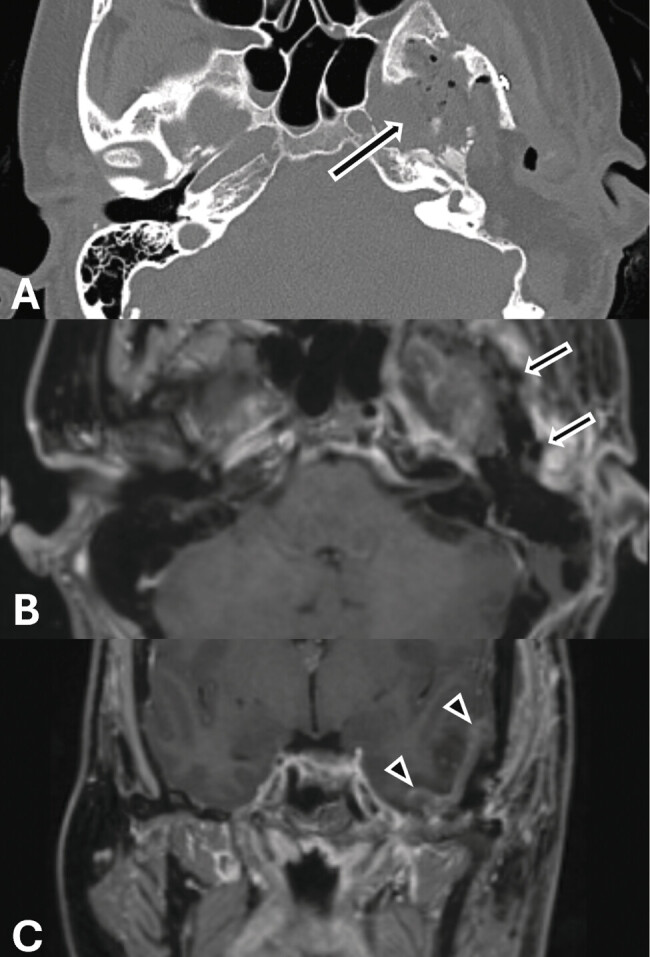
( **A** ) Axial computed tomography (CT) through the bilateral temporal bones demonstrates postoperative resolution of previously noted sclerosis and hyperostosis (solid arrow). ( **B** ) Axial T1-weighted postcontrast fat-saturated image demonstrates left lateral skull base resection (solid arrows). ( **C** ) Coronal T1-weighted postcontrast fat-saturated image demonstrates expected postsurgical changes and a small amount of extra-axial fluid along the left temporal convexity (arrowheads). No findings suggest residual disease. Note the normal magnetic resonance imaging appearance of the right side.

The postoperative course was initially uneventful, but three months later, her left ear fullness, pain, and pressure recurred, with her otologic examination reverting to preoperative status. Despite the placement of a pressure equalization tube, she continued to experience left-sided temporal headaches, pulsatile tinnitus, ear fullness, and conductive hearing loss with a persistent ABG of 33 dB, which altogether limited her ability to fulfill academic and professional obligations. After discussion of her case at a multidisciplinary conference, she elected to undergo a temporal craniotomy, subtotal petrosectomy (STP), resection of the meningioma, and placement of an abdominal fat graft. Despite the likelihood of worsening hearing loss after STP, this approach was chosen to prioritize tumor resection and pain relief, in line with the patient's goals. The neurosurgical approach involved a left temporal craniotomy, dural-based soft tissue tumor excision, and bony removal of the middle fossa floor centered over the temporomandibular joint, with medial extension to the foramina spinosum, ovale, and rotundum. After surgery, she developed temporal encephalopathy, which resolved without intervention. Postoperative CT ( Fig. 6A ) and MRI ( Fig. 6B, C ) demonstrated resection in the left mastoid region. Audiometric testing following STP demonstrated an air PTA of 81 dB, an ABG of 58 dB, and a WRS of 69%. At 6-month follow-up, she reported significant pain improvement. Eleven months later, with her pain remaining stable but maximal conductive hearing loss persisting, a Cochlear Baha Connect system was placed to support her professional responsibilities and daily activities. She has undergone follow-up evaluations at one and four months following Baha placement to assess soft tissue adaptation, audiologic outcomes, and device function, with plans for continued long-term monitoring.

## Discussion

We report a patient with a left temporal bone en plaque meningioma who experienced significant pain relief following surgical resection. While hearing loss and otorrhea are common presenting symptoms of temporal bone meningiomas, pain is less frequently reported, and its underlying mechanisms are not well understood. The limited exploration of pain may be due to the variety of underlying mechanisms involved. It is known that en plaque meningiomas can cause symptoms through mass effect on adjacent structures, the narrowing of cranial nerve foramina such as Meckel's cave, or through local inflammation. 6 Potential sources of pain in this case include significant osteitis of the middle fossa floor, geniculate neuralgia, or meningeal inflammation surrounding the en plaque tumor component.

The variability in pain presentation among patients with temporal bone en plaque meningiomas is evident from individual case studies. As previously reported, pain remains a relatively uncommon presenting symptom even among temporal bone lesions. Ayache et al 5 reported a 49-year-old woman with a temporal bone en plaque meningioma who experienced headaches and symptoms suggestive of trigeminal neuralgia, supporting the possibility that pain may arise secondary to adhesion and compression of adjacent neural structures. Notably, during our patient's outside mastoidectomy, a lesion adherent to the facial nerve was noted, suggesting that the proximity of the tumor and/or related inflammation to the facial nerve could have been a source of pain. Evidence suggests that, due to its mixed motor and sensory innervation, compression of the facial nerve by skull base tumors can lead to varying degrees of dysfunction, 7 as seen in this case with severe otalgia and debilitating headaches. The initial pain may have persisted due to the tumor's proximity to the geniculate ganglion, which was not approached from the mastoid. Subsequent resection of the tumor in the epitympanum and over the geniculate from the middle fossa approach may explain our patient's significant postoperative pain improvement.

The limited number of reported temporal bone en plaque meningiomas reflects the variation in clinical presentation and management strategies. To date, only 25 other cases have been reported in the literature. 1 5 8 9 10 11 12 13 14 15 16 17 18 Pain was reported in four cases, 5 11 but surgery was pursued in only one—the case described by Ayache et al. 5 Despite incomplete resection, this patient experienced complete resolution of pain, further supporting the notion that factors beyond local inflammation may be at play. The other three patients were treated medically, possibly due to surgical risks, preexisting comorbidities, or tumor location. Among the remaining 21 temporal bone en plaque meningiomas without reported pain, treatment strategies included surgery in 10 cases, radiation therapy in two, and conservative management in eight, whereas one case lacked documentation of the management plan. 1 5 8 9 10 12 13 14 15 16 17 18 Among those with documented follow-up, no surgical complications were reported, yet the decision to pursue surgery in these tumors remains complex.

Understanding the mechanism of pain in en plaque meningiomas is crucial for guiding surgical decisions. Still, even in the presence of symptoms, the management of these tumors remains debated. Extensive bone invasion often complicates total resection, promoting a preference for conservative management. 5 While it could be argued that discomfort may not justify the risk of postoperative cranial nerve dysfunction, Vrionis et al 1 supported aggressive resection in young patients with progressive symptoms despite this risk. Our patient's significant relief from debilitating pain, despite the anatomical challenges, highlights that surgical intervention may offer symptomatic benefit in select cases. However, outcomes, particularly regarding pain, remain variable and should be considered carefully when counseling patients.

## Conclusion

Pain may contribute to the decision to pursue surgical resection in temporal bone en plaque meningiomas, but its resolution remains unpredictable. To enhance patient outcomes and quality of life, a more systematic approach to pain management in temporal bone en plaque meningiomas is needed.
